# Fetal magnetic resonance imaging of lumbar spine development in vivo: a retrospective study

**DOI:** 10.1007/s00381-022-05645-x

**Published:** 2022-08-16

**Authors:** Xing Yin, Xin Zhao, Lin Lu, Liying Zhang, Qingna Xing, Rui Yuan, Zhijun Niu, Linlin Zhang

**Affiliations:** 1 Department of Radiology, The Third Affiliated Hospital of Zhengzhou University, Zhengzhou, China; 2 Department of Ultrasound, The Third Affiliated Hospital of Zhengzhou University, Zhengzhou, China; 3 Department of Obstetrics and Gynecology, The Third Affiliated Hospital of Zhengzhou University, Zhengzhou, China; 4 Department of Laboratory Medicine, The Third Affiliated Hospital of Zhengzhou University, Zhengzhou, China

**Keywords:** Fetus, Magnetic resonance imaging, Spine

## Abstract

**Objective:**

The aim of this study is to describe MR imaging appearances of the fetal lumbar spine in vivo at different gestational ages (GAs).

**Methods:**

This retrospective study was approved by the Third Affiliated Hospital of Zhengzhou University. We collected MR images and clinical data of 93 fetuses in our hospital. All the MR images were obtained by 3-T MR. All had the mid-sagittal plane of steady state free precession sequence (Trufi) of the lumbar spine, which could show the lumbar vertebra and conus medullaris (CM). Regression analysis was made between GA and heights of lumbar vertebral body ossification center (LVBOC), lengths of LVBOC, and heights of intervertebral gap (IVG).

**Results:**

There were good linear correlations between the heights of LVBOC and GA (*P* < 0.001), lengths of LVBOC and GA (*P* < 0.001), and heights of IVG and GA (*P* < 0.001).

**Conclusion:**

We showed the different development of each LVBOC and IVG which caused the difference of the shape of LVBOC and IVG.

## Introduction

Because of more detailed information obtained by the MR, fetal MR could be very useful for the diagnosis and treatment of fetal disease [1–5]. Before evaluating the fetal spine, it is important to understand the development of fetal spine at different GAs [4, 6]. We find few of published dates on the appearances of vertebrae and intervertebral space, and most of them were postmortem [7–13]. The aim of this study is to describe MR imaging appearances of the fetal lumbar spine in vivo at different GAs [14, 15].

## Material and methods

This retrospective study was approved in the Third Affiliated Hospital of Zhengzhou University. Between September 2017 and March 2021, 353 fetuses were examined in our hospital which due to the abnormality of the central nervous system indicated by ultrasound, which included mild ventriculomegaly and abnormal width of the cavum septum pellucidum, and their central nervous system abnormalties were confirmed by the MR, and none of them had other positive findings in ultrasound and MR, and all of them had MR images of fetal head and spine. Mild ventriculomegaly (ventricle width of 10–12 mm) without other systemic malformations is usually considered to have normal neurodevelopmental processes [16]. Without of aneuploidy and other associated fetal abnormalities, the neurodevelopment appears to be normal in the abnormal width of the CSP prenatally [17]. Because of the prognosis, these fetuses were considered to be low risk and selected in the study. Reasons for exclusion included blurry images due to fetal movement (*n* = 138), no mid-sagittal image of fetal lumbar spine images (*n* = 107), and inability to identify the L5 position (*n* = 15), and 93 fetuses, from 22 to 37 gestational weeks (median, 27.1 gestational weeks) were collected in our study in the end. The GA was estimated by the date of woman’s last menstrual period (*n* = 82) or the assessments made by sonography (*n* = 11).

All the MR images were obtained by 3-T MR (Skyra, Siemens Medical Systems, Germany) with an eight-channel body surface coil. Pregnant women were placed in the supine position, and those who were difficult to examine in the supine position could be placed in the left lateral position. There was no fetal sedation, and if fetal movement was obvious, the pregnant woman would have rest, then we continued to examine. Three-plane imaging of T2WI of fetal spines was acquired. All had the mid-sagittal plane of steady-state free precession sequence (Trufi, repetition time/echo time = 740/2.4 ms; field of view, 35 × 45 cm; thickness, 3 mm; − 0.6 mm gap; voxel size, 1.1 × 1.1 × 3.0 mm; FA, 58°; NEX, 1) of the lumbar spine, which could show the lumbar vertebra and CM. The other sequences consisted of the T2WI half-Fourier acquisition single-shot turbo spin echo sequence (HASTE, repetition time/echo time = 1400/63 ms; field of view, 40 × 40 cm; thickness, 4 mm; 0.8-mm gap; FA, 120°; NEX, 1), T1WI fast-low angle shot (repetition time/echo time = 120/2.4 ms; field of view, 35 × 40 cm; thickness, 4 mm; 0.8 mm gap; FA, 70°; NEX, 1), and susceptibility-weighted imaging (SWI, repetition time/echo time = 10/5 ms; field of view, 35 × 35 cm; thickness, 3 mm; 0 mm gap; FA, 15°; NEX, 1). All the MR images were transferred to PACS (Synapse, Fujifilm medical system, Japan).

**Fig. 1:**
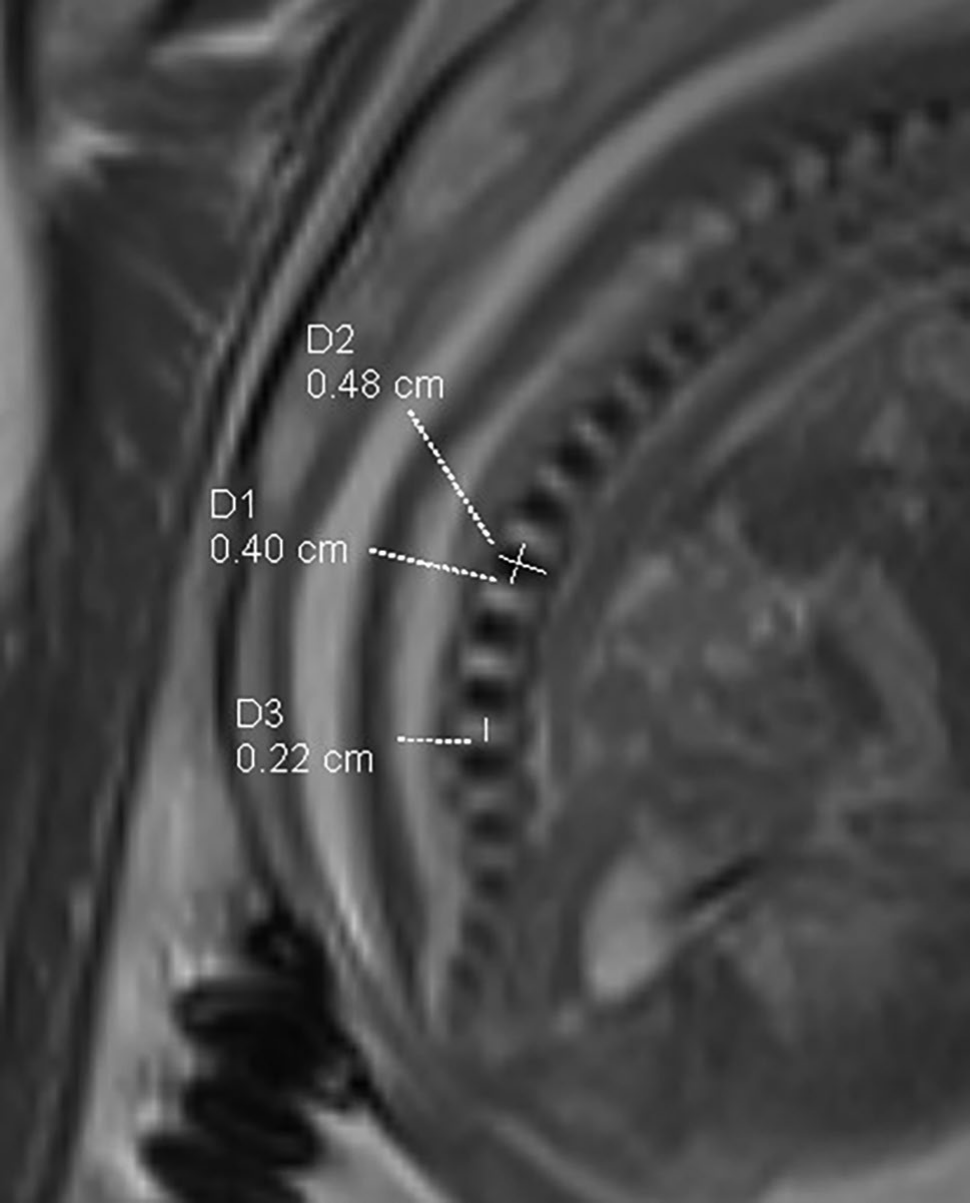
A total of 26 gestational weeks, Trufi. Measuring of height and length of L1 vertebral ossification body center, and height of L3–4 IVP

All the measurements were carried on the mid-sagittal plane of Trufi (Fig. 1). Lumbar ossification centers are defined as hypointensity in the spinal vertebral region of the Trufi sequence. Localizing the iliolumbar ligament was been used to determine the lumbar vertebral level, and counting down from C2 might be a supplement, although more difficult since cervical vertebral ossification centers were extremely small and it was difficult to get the mid-sagittal images of the whole spine. If localization was still difficult, the case would be removed. Before independent measurements were performed by two pediatric radiologists (10 years of clinical work experience in our hospital), they had to make an agreement on the location of the L5. The mean of the two measurements was then used for further analysis. SPSS 20.0 was used for the data analysis. Simple linear regression analysis was made between GA and heights of lumbar vertebra body ossification center (LVBOC), lengths of LVBOC, and heights of intervertebral gap (IVG).

## Results

**Fig. 2:**
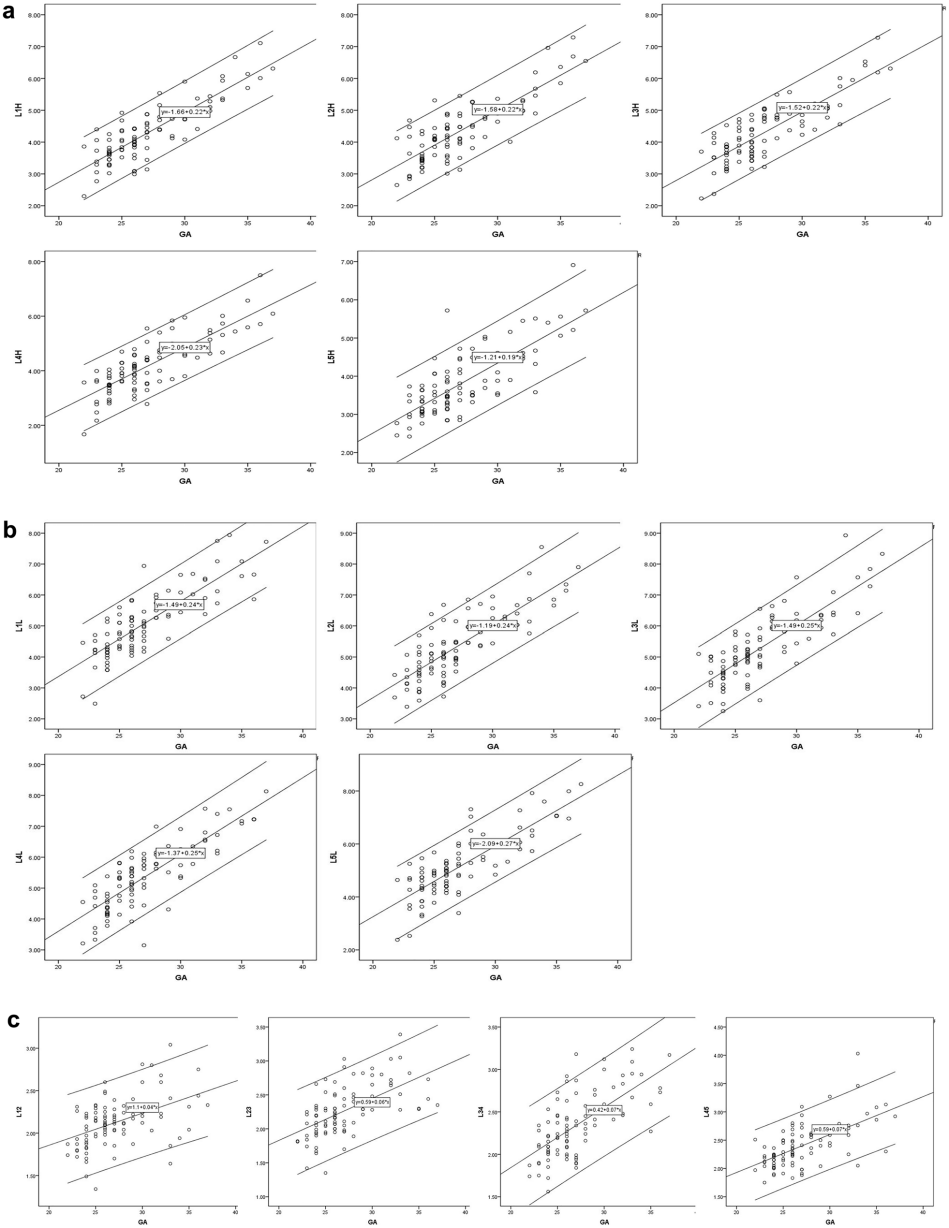
Scatter plot shows a linear relation between the heights of LVBOC and GA (**a**, L1H, L2H, L3H, L4H, and L5H stand for heights of LVBOC of L1, L2, L3, L4, and L5), the lengths of LVBOC and GA (**b**, L1L, L2L, L3L, L4L, and L5L stand for lengths of LVBOC of L1, L2, L3, L4, and L5), and the heights of IVP and GA (**c**, L12, L23, L34, and L45 stand for heights of IVP of L12, L23, L34, and L45)

There was a good inter-rater agreement on the measurements of heights of LVBOC, lengths of LVBOC, and heights of IVG (*P* < 0.001). There were good linear correlations between the heights of LVBOC and GA (*P* < 0.001), lengths of LVBOC and GA (*P* < 0.001), and heights of IVG and GA (*P* < 0.001). The results of the correlation are shown in Tables 1, 2, and 3. Scatter plots are shown in Fig. 2a, b, and c.

**Table 1:** Linear correlation between the heights of LVBOC and GA

	*R*^2^	*P*	Constant	Slope
Height of L1	0.716	< 0.001	− 1.660	0.220
Height of L2	0.667	< 0.001	− 1.580	0.219
Height of L3	0.681	< 0.001	− 1.516	0.215
Height of L4	0.646	< 0.001	− 2.049	0.23
Height of L5	0.585	< 0.001	− 1.209	0.185

**Table 2:** Linear correlation between the lengths of LVBOC and GA

	*R*^2^	*P*	Constant	Slope
Length of L1	0.670	< 0.001	− 1.488	0.243
Length of L2	0.654	< 0.001	− 1.193	0.241
Length of L3	0.652	< 0.001	− 1.488	0.250
Length of L4	0.674	< 0.001	− 1.366	0.248
Length of L5	0.658	< 0.001	− 2.092	0.267

**Table 3:** Linear correlation between the heights of lumbar IVG and GA

	*R*^2^	*P*	Constant	Slope
Height of L1–2	0.210	< 0.001	1.102	0.038
Height of L2–3	0.333	< 0.001	0.591	0.062
Height of L3–4	0.431	< 0.001	0.423	0.071
Height of L4–5	0.373	< 0.001	0.587	0.067

## Discussion

**Fig. 3:**
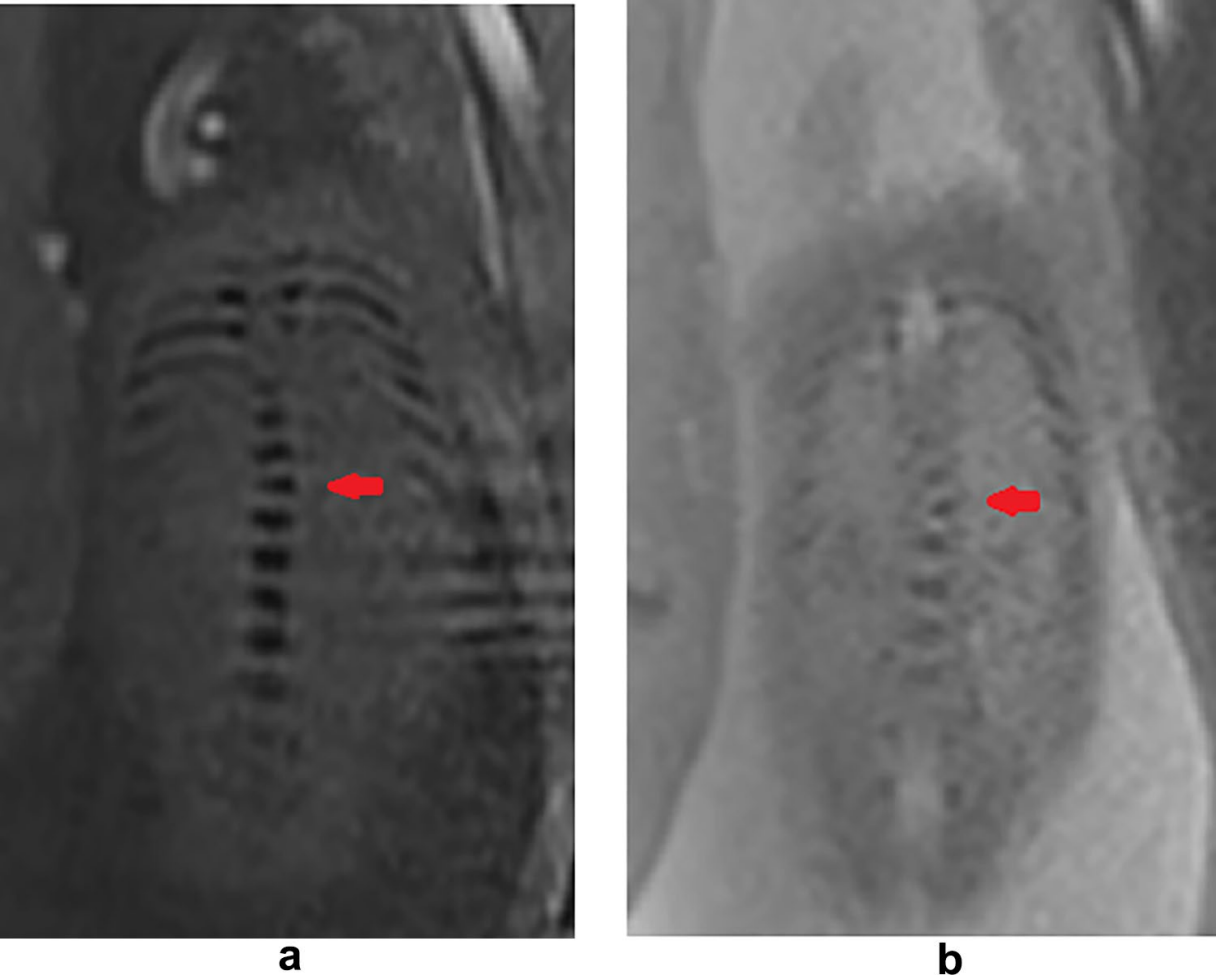
24 weeks of gestation, SWI (**a**) and Trufi (**b**) showed that T10 (red arrow) was a wedged vertebra, and the spine was slight scoliosis. The chromosome karyotyping and chromosome microarray analysis report fetal trisomy 18 (not included in study cases)

The development of the spine includes 6 periods: (1) formation of the somitic mesoderm and notochord, (2) formation of the somites, (3) formation of the dermomyotome and sclerotome, (4) the membranous phase, (5) vertebral chondrification, and (6) vertebral ossification [18, 19]. The ossification of the vertebra begins from the thoracolumbar junction [18]. Generally, at present, fetal MR is imaged after 18 gestational weeks, and at this time, all the lumbar ossification centers could be seen [1, 20]. Many diseases can cause changes in the height of the vertebral body, such as the wedge vertebra (Fig. 3). Wedge vertebra is a cause of scoliosis. Wedge vertebra usually does not cause obvious scoliosis [1, 20], but the height of the vertebral body usually decreases on one side [19].

**Fig. 4:**
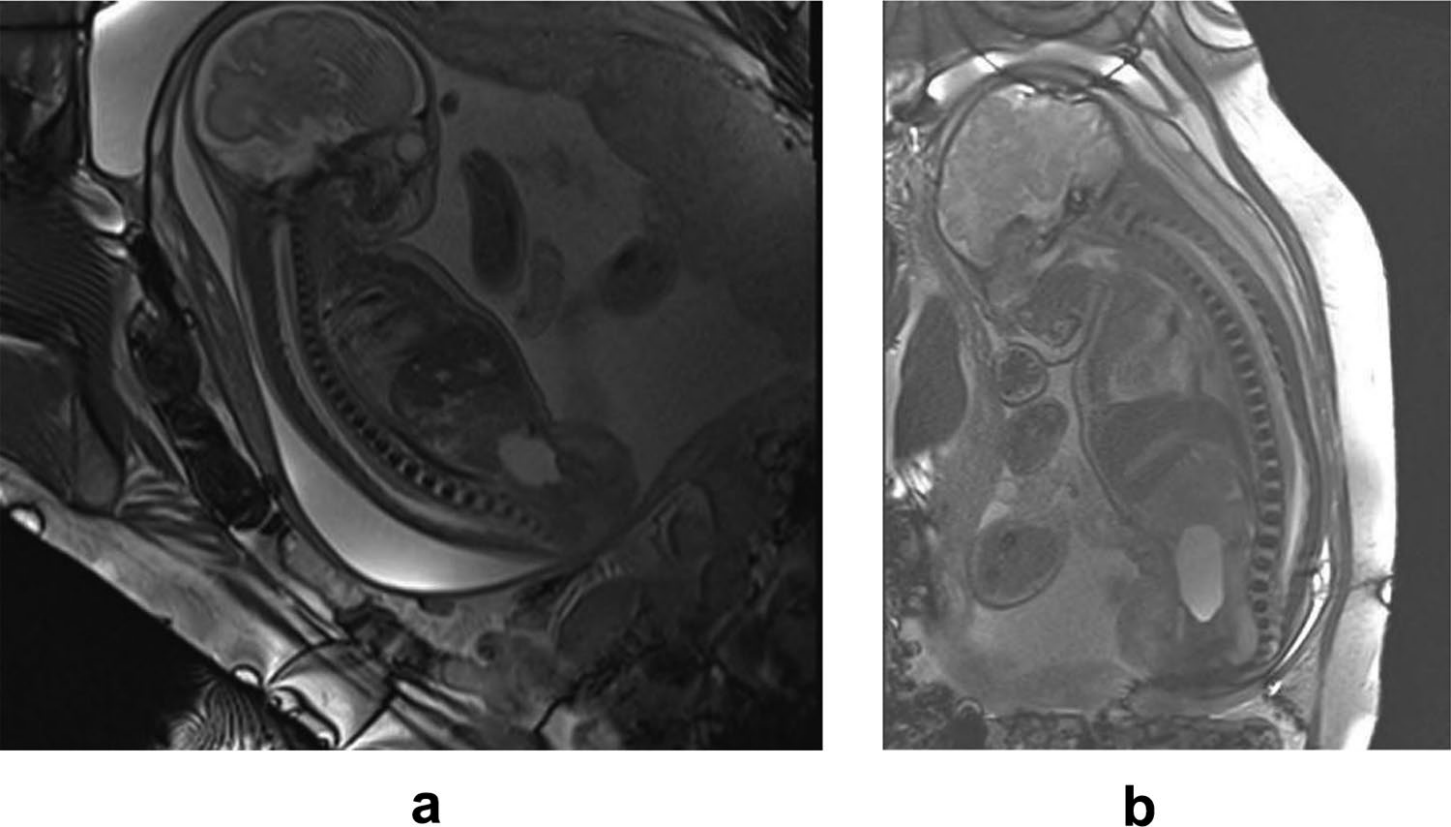
The same fetus was examined at 28 (**a**) and 36 (**b**) weeks of gestation. Trufi images of the mid-sagittal plane of the spine showed the development of heights and lengths of LVBOC and heights of IVP. Note the development of the cerebral cortex

**Fig. 5:**
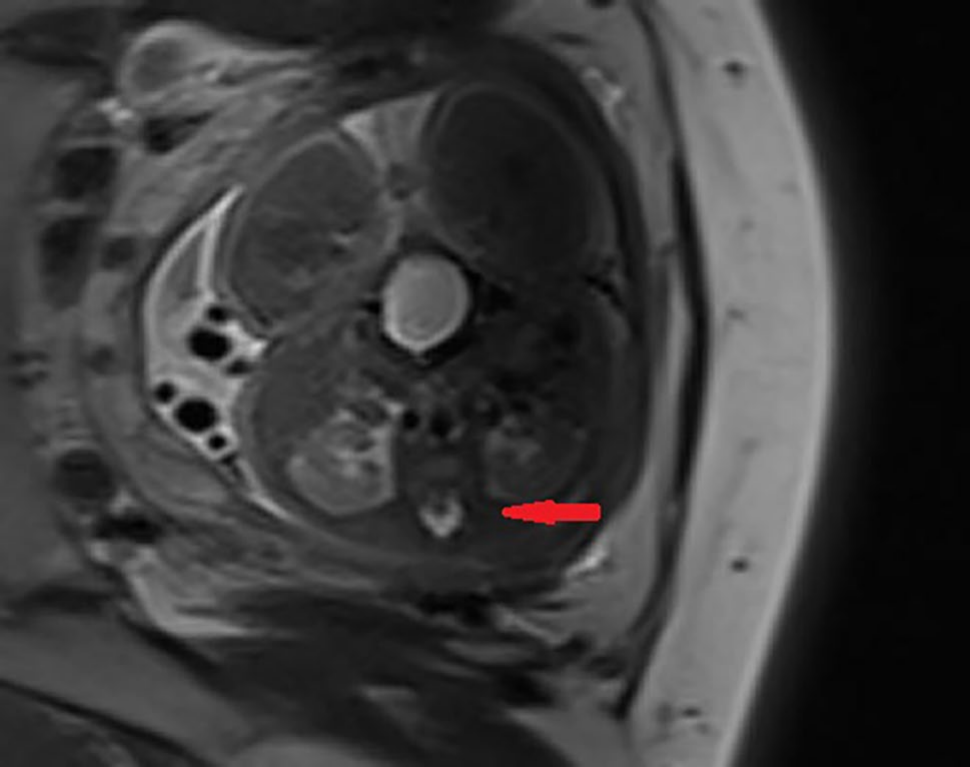
Half-Fourier acquisition single-shot turbo spin echo sequence (HASTE) of lumbar spine shows the spinal canal, cerebrospinal fluid, and lumbar spinal cord (red arrow)

The steady-state free precession sequence (Fig. 4) is the major sequence for the fetal spine MR, which shows the vertebral ossification center better than the single-shot turbo spin echo sequence, and the contrast signal to noise ratio between the vertebral ossification center and intervertebral space is better [1, 21–23]. The single-shot turbo spin echo sequence (Fig. 5) shows better of the soft tissue and spinal cord [1, 21–23]. SWI sequence (Fig. 3a) shows vertebral ossification center is better than the steady state free precession sequence, especially MIP images can be performed to display ossification centers of the spine and thorax, but the soft tissue and spinal cord cannot be displayed [4]. Since the SWI images are often blurry because of fetal movement and maternal respiration, they are only used as a secondary means in our hospital [4].

Each centrum is fused by the inferior half and the superior half of the sclerotomes, so the intersegmental vessels are located in the center of the vertebral bodies [4]. On fetal MR T2WI, the vertebral ossification center is usually shown as a hypointensity in the spinal vertebral region [24]. The hyperintensity in the central vertebral ossification center due to the intersegmental vessels is rare in MR in vivo. The morphology of the fetal vertebral ossification center can be manifested as biconvex, bullet-like, rectangular hypointensity on T2WI images [24]. The intervertebral disc showed uniform hyperintensity on T2WI on MR. In the axial images, the ossification centers of the lumbar vertebrae appear as round-shaped T2 hypointensity. The ossification centers of lumbar vertebral arches are distributed on both sides and no fusion occurred [6]. Oblique axial scanning was usually required to observe the ossification center of the vertebral body and the bilateral vertebral arches.

Localization of lumbar vertebral is difficult in some cases in vivo examinations. Due to the influence of maternal respiration and fetal movement in the examination, it cannot locate the lumbar vertebral in sagittal position by 12th rib in coronal position or L5 nerve in axial image [25]. Localizing the iliolumbar ligament is the most commonly used method in the sagittal position [26]. This method is not very accurate. It is more accurate to count down from C2, and as discussed previously, it is more difficult because of the small cervical vertebral ossification centers, and it was difficult to get the mid-sagittal images of the whole spine.

In our study, we find the linear relationship between the heights of LVBOC and GA, lengths of LVBOC and GA, and heights of IVG and GA. The slopes of the linear relationship between heights of LVBOC from L1 to L4 and GA are very similar, and the slop of L5 is the minimum. The slopes of the linear relationship between the lengths of every LVBOC and GA are very similar. Due to the asynchrony of their development, the size and shape of the vertebral bodies are ultimately different [11, 12, 18, 27]. The height slope of the lumbar IVG is inconsistent, indicating that their development is not synchronized, L3-4 grows the fastest and L1–2 the slowest [12, 18].

There are limitations to our study. Since this is a retrospective study and no MR follow-up was performed in our cases on the spine after birth, we are unable to evaluate the lumbar spine after birth yet. Further studies to evaluate the association between prenatal and postnatal study, as well as the long-term follow-up, are needed, and we intend to use the 3D software to describe values of lumbar vertebra based on the 3D sequence in the future.

## Conclusion

We demonstrate a good linear relationship between the development of the lumbar spine and gestational age in vivo by MR.
